# PROFESSOR JULIO CEZAR UILI COELHO. FORMER PRESIDENT OF THE BRAZILIAN COLLEGE OF DIGESTIVE SURGERY

**DOI:** 10.1590/0102-672020230047e1765

**Published:** 2023-10-13

**Authors:** Antonio Carlos Ligocki Campos

**Affiliations:** 1 Universidade Federal do Paraná, Department of Surgery – Curitiba (PR), Brazil

**Figure:**
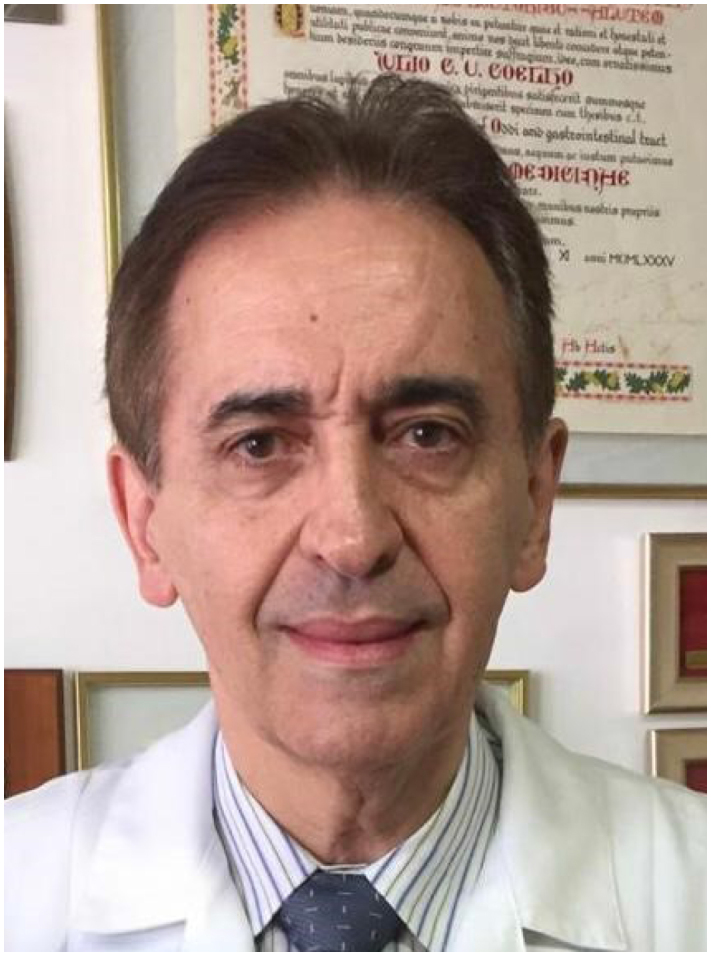


I accepted the honorable invitation to write this editorial about Professor Julio Cezar Uili Coelho with a dubious feeling: if, on the one hand, our fraternal friendship of more than 40 years together facilitates this task, on the other hand, I am faced with the Herculean challenge of summarizing the academic trajectory of one of the greatest professionals in Digestive Surgery in our country and in the world. Very few professors can be compared to Prof. Julio Coelho in terms of scientific publications, both quantitatively and qualitatively, in the impeccable university career and enviable academic training pursued in Brazil and, later, on both sides of the Atlantic.

The third son of Mr. Fabio and Mrs. Nefege, Julio was born in the north of the state of Paraná, in the small town of Ibiporã, on March 22, 1953. His father came from the city of Muriaé (state of Minas Gerais) and his mother, from the district of Batista Botelho (state of São Paulo). Its family roots originate in Italy, Portugal, Spain and Syria, a reflection of the exuberant miscegenation of our country, this true melting pot that enriches us so much.

As a child, Julio moved to Maringá (PR), where he started elementary school and, later, the family permanently settled in Curitiba (capital of PR). He studied Medicine at Universidade Federal do Paraná (UFPR), where he graduated in 1976.

During college, Julio stood out as an excellent student, being approved in tutoring services and internships within and outside UFPR. From 1977 to 1979, he was a resident physician in the discipline of Gastrointestinal Surgery at UFPR, under the coordination of Prof. Giocondo Villanova Artigas, who played an important role in his education.

After completing his medical residency, seeking to improve his university education, he entered the University of Illinois, in Chicago (USA), under the supervision of Professors Lloyd M. Nyhus and Bernard Sigel, from 1979 to 1982, where he worked as a resident in general surgery and later as a clinical fellow. He also had the opportunity to attend the master's and PhD programs at this prestigious university, where he completed his master's thesis in 1980 and his PhD in medicine in 1982. During this period of intense scientific activity, he participated in several pioneering and world-renowned studies on the use of perioperative ultrasound.

In November 1980, he was approved in a public tender to become assistant professor in the discipline of Gastrointestinal Surgery at UFPR, a position he assumed in April 1982, when he returned from the USA. For holding a PhD in medicine, he was subsequently promoted to associate professor at the institution.

From August 1983 to June 1985, he left UFPR to improve himself, at the postdoctoral level, at the University of Texas, where, under the supervision of Prof. Frank G. Moody, he was a visiting assistant professor in the Department of Surgery, also with intense scientific activity and dozens of cutting-edge publications in the area of gastrointestinal motility.

With the result of his research on gastrointestinal motility, he obtained a Doctor of Medicine degree from the University of Limburg (Maastricht, Netherlands) in 1985. Later, he received a scholarship and the fellow title of the Alexander von Humboldt Foundation and worked as a visiting professor at the Heidelberg University, where he received the title of Doctor of Medicine after passing the Rigorosum examination and his thesis being approved with *Magna Cuminatum* academic distinction. In summary, Julio Coelho has three PhDs from leading universities in Europe and the USA, all of which were revalidated in Brazil.

In early 1986, he resumed his duties as associate professor of Gastrointestinal Surgery at UFPR. In July of the following year, he was approved in a public tender for Titular Professor at the Department of Surgery at the School of Medicine of Ribeirão Preto, Universidade de São Paulo.

With funding for research from the Funding Agency for Studies and Projects (*Financiadora de Estudos e Projetos* – FINEP) and scholarships from the National Council for Scientific and Technological Development (CNPq), he formed the Gastrointestinal Motility Laboratory of the Gastrointestinal Surgery discipline, of which he assumed the coordination in 1989, a position he holds to this day.

In 1990, he edited the book entitled *Aparelho digestivo. Clínica e cirurgia* [“Gastrointestinal system. Clinic and surgery”], with 1,400 pages, 174 chapters and 270 collaborators, with the participation of 89 professors from 21 countries. The second edition was published in 1996, followed by the third in 2005, and the fourth in 2012^1^. This book, the best seller in Brazil in this area, is adopted by several universities in the country. In 2009, he published the *Manual de clínica cirúrgica. Cirurgia geral e especialidades* [“Handbook of surgery clinics. General surgery and specialties”], in two volumes, totaling 2,746 pages, 305 chapters, and 361 collaborators from 26 countries^2^.

In 1990, he was approved in the first place in the public tender for full professor of the Department of Surgery at UFPR, becoming, at the time, the youngest full professor of surgery in Brazil. In September 1991, he successfully performed the first liver transplantation in Paraná, at Hospital de Clínicas of UFPR. This program is still active to date, with hundreds of adult and pediatric transplant recipients^3,5,6^.

His scientific production is enviable. Julio Coelho has more than 400 published works, of which 124 abroad, 28 editorials and hundreds of studies presented in national and international congresses, in addition to over one hundred book chapters and abstracts published^4^.

He participated in 367 round tables, symposiums, colloquiums, panels, and videosurgery presentations and delivered 328 lectures in several Brazilian states and 19 abroad. He was a visiting professor at 11 universities in the USA, Europe, and Asia and supervised 31 master's and PhD students. He participated in 84 university committees and medical societies, including: president of the Brazilian College of Digestive Surgery (*Colégio Brasileiro de Cirurgia Digestiva* – CBCD) in the 2005-2006 biennium, Chairman of the Board of Paraná of the Brazilian College of Surgeons (*Colégio Brasileiro de Cirurgiões*); president of the Paraná Society of Gastroenterology and Nutrition (*Sociedade Paranaense de Gastroenterologia e Nutrição*) and of the Paraná Society of Laparoscopy (*Sociedade Paranaense de Cirurgia Laparoscópica*).

During his tenure as president of the CBCD, he was responsible for preparing the *Manuais de orientação para o paciente* [“Guidance Handbooks for Patients”]. These handbooks were created to help physicians guide their patients regarding the condition to be treated, symptoms, diagnosis, treatment, complications, care, and prevention. They also include a free and informed consent, in which patients attest that they received all the instructions from their surgeons before any procedure and that all their doubts were clarified. Surgeons acquire the handbooks and make them available to patients in their offices for consultation on the following topics: gallstones, gastroesophageal reflux disease, morbid obesity, stomach cancer, and inguinal hernia. The handbooks are periodically reviewed and continue to be acquired by CBCD members, as they are recognized for the importance of clarifying patients about their conditions.

Julio Coelho received 39 awards and honors in Brazil and abroad, including: honorable mention from the Chicago Society of Surgery (USA), in 1981; *Cirurgião Jovem* [“Young Surgeon”] Award from the Brazilian College of Surgeons (1988); National Gastroenterology Award (1990); Science and Technology Award of the State of Paraná (1991); Award from the Surgical Assembly of the Brazilian College of Surgeons (1992); Gold Medal from the Oswaldo Cruz Foundation of the Brazilian Ministry of Health (2002); and Paraná Medical Association Award — Distinction in Medicine in Teaching and Research (2007). He is also a member of the Paraná Medicine Academy, of the editorial board of 15 national and international medical journals, and has participated in 236 examination boards for university professors and thesis defenses at several Brazilian universities.

Julio married Célia Zardo Coelho at the end of college education and they had two daughters, who were born in the USA: Caroline, in Chicago, and Christine, in Houston. Both of them are lawyers. Caroline gave him two of his greatest treasures: the grandsons Nicholas and Anthony. Julio married Karla Christina Maron Coelho in 1991, over 30 years ago, and shares a stable and peaceful life with her. She is his refuge after his exhausting surgical journeys. Karla is also his companion on the many exotic trips they often take.

For leisure, in addition to the exotic trips he usually takes and the countless cruises around the world, Julio has an enviable collection of more than 500 statues, mostly African and Asian, as well as countless Greco-Roman ones, all cataloged and kept with great care as a souvenir of his travels. Currently, Julio needs to share the time dedicated to his statues with his grandchildren, who constantly challenge him to overcome his restricted football skills.

Finally, I feel privileged to enjoy his friendship, share his knowledge and the paths taken in the Department of Surgery at UFPR alongside such an illustrious and emblematic professional, whose character is solidly forged in ethics, seriousness, competence, and dedication to the academic life.

Antonio Carlos Ligocki Campos is President of the Brazilian College of Digestive Surgery (2023–2024).
